# Supplementary material for: A systematic review and meta-analysis of magnetic resonance and computed tomography enterography in the diagnosis of small intestinal tumors
Source: PeerJ. 2023 Dec 21;11:e16687. doi: 10.7717/peerj.16687 (PMC10749088; doi:10.7717/peerj.16687)
Supplement: Supplemental Information 3 [file peerj-11-16687-s003.docx]

**The rationale for conducting the systematic review/meta-analysis:**

The small intestine is coiled and overlapped in the abdominal cavity, with significant movement, which inconveniences the imaging examination. Gastrointestinal barium meal has always been the first choice for examination, and CT enterography plays an increasingly prominent role in the diagnosis and evaluation of intestinal diseases with the development and improvement of CT technology, which clearly shows the lesions of the intestinal wall and intestinal cavity, with easy operation and more acceptances to patients. MR has no ionizing radiation than CT, which is appropriate for pregnant women and children who are not suitable for CT examination, and its high soft tissue resolution could accurately display anatomical details of patients' internal, parietal, and external small intestines and qualitatively analyze SIT. For example, T1-weighted SIT or lipomas containing fat components show high signals and T2-weighted hemangiomas show obvious high signals. The application of MR and CT enterography in diagnosing SIT shows inconsistent conclusions and controversial clinical indicators in each study. Therefore, this study objectively evaluates the value of MR and CT enterography in diagnosing SIT through more comprehensive retrieval and screening and provides more bases for selecting and formulating clinical treatment options. It has a reasonable prospect of clinical application.
